# Universal immunity to influenza must outwit immune evasion

**DOI:** 10.3389/fmicb.2014.00285

**Published:** 2014-06-12

**Authors:** Sergio Quiñones-Parra, Liyen Loh, Lorena E. Brown, Katherine Kedzierska, Sophie A. Valkenburg

**Affiliations:** ^1^Department of Microbiology and Immunology, The University of Melbourne at the Peter Doherty Institute for Infection and Immunity, ParkvilleVIC, Australia; ^2^Centre for Influenza Research and School of Public Health, The University of Hong KongHong Kong, China

**Keywords:** influenza viruses, T cells memory, antibodies, viral escape mechanisms, vaccine design

## Abstract

Although an influenza vaccine has been available for 70 years, influenza virus still causes seasonal epidemics and worldwide pandemics. Currently available vaccines elicit strain-specific antibody (Ab) responses to the surface haemagglutinin (HA) and neuraminidase (NA) proteins, but these can be ineffective against serologically-distinct viral variants and novel subtypes. Thus, there is a great need for cross-protective or “universal” influenza vaccines to overcome the necessity for annual immunization against seasonal influenza and to provide immunity to reduce the severity of infection with pandemic or outbreak viruses. It is well established that natural influenza infection can provide cross-reactive immunity that can reduce the impact of infection with distinct influenza type A strains and subtypes, including H1N1, H3N2, H2N2, H5N1, and H7N9. The key to generating universal influenza immunity through vaccination is to target functionally-conserved regions of the virus, which include epitopes on the internal proteins for cross-reactive T cell immunity or on the HA stem for broadly reactive Ab responses. In the wake of the 2009 H1N1 pandemic, broadly neutralizing antibodies (bnAbs) have been characterized and isolated from convalescent and vaccinated individuals, inspiring development of new vaccination techniques to elicit such responses. Induction of influenza-specific T cell responses through vaccination has also been recently examined in clinical trials. Strong evidence is available from human and animal models of influenza to show that established influenza-specific T cell memory can reduce viral shedding and symptom severity. However, the published evidence also shows that CD8^+^ T cells can efficiently select immune escape mutants early after influenza virus infection. Here, we discuss universal immunity to influenza viruses mediated by both cross-reactive T cells and Abs, the mechanisms of immune evasion in influenza, and propose how to counteract commonly occurring immune-escape variants.

## Introduction

Immunization is the most cost effective public health measure to prevent the spread of infectious diseases. Influenza causes seasonal epidemics as well as periodic global pandemics due to the introduction of novel strains and sporadic outbreaks from animal reservoirs. During the 2009 H1N1 pandemic, the overall infection rate was 10%, although the infection rate rose to 43% in school-aged children (Wu et al., 2010). Yet, an influenza vaccine has been available since the 1940's.

Current influenza vaccines predominantly mediate protection by eliciting neutralizing Ab responses to epitopes on the head region of the virion surface glycoprotein, HA, and also to the NA. The traditional trivalent influenza vaccine (TIV) is based on either inactivated whole/subvirion virus or detergent-disrupted virions, and is administered as an intramuscular injection to elicit systemic Ab responses. Alternatively, the live attenuated influenza vaccine (LAIV), is given intranasally. The LAIV vaccine does not boost T cell immunity in adults (He et al., 2006), but does improve protection by eliciting Ab responses locally in the respiratory tract. Notably, LAIV in children is able to establish influenza-specific T cell responses, possibly due to their naïve infection status (He et al., 2006). Whilst LAIV has increased protection compared to TIV (Monto et al., 2009), TIV has much wider use due to a greater number of manufacturers and constrained use of LAIV in the elderly and very young.

Originally, the influenza vaccine was a monovalent preparation, based on the common circulating strain of influenza A virus (IAV) and was swiftly updated to include an influenza B virus when these were recognized in the 1940's. Since the 1970's, a trivalent vaccine has been used to provide coverage for H1N1, H3N2, and influenza B virus, which now co-circulate. Even more recently, in 2013, the vaccine has become available in a quadrivalent form incorporating both Victoria and Yamagata lineages of influenza B. Thus, the number of vaccine strains has progressively been increased from a single strain to four, to provide broader protection and overcome the diversity of multiple influenza virus subtypes and lineages. Nevertheless, within each subtype or lineage, constant antigenic drift gives rise to new and unpredictable antigenic variants that are not necessarily represented in the vaccine. Mismatch between the vaccine strain, predicted on the basis of antigenically novel isolates circulating in the previous winter in the opposite hemisphere, and the actual strain that emerges in the current winter can result in significant loss of vaccine effectiveness.

The mechanism of action of current vaccines, which mediate protection by induction of neutralizing Ab responses to the rapidly changing head of the HA protein, renders it ineffective after a few years at best, once all the antigenic regions on the head of the HA have mutated in response to pre-existing Ab. Inactivated vaccines are also very poor inducers of CD8^+^ T cell responses, presumably because of inefficient uptake and priming by appropriate antigen presenting cells (APCs). There is evidence from animal models that TIV vaccination can actually inhibit the induction of cross-reactive T cell responses (Bodewes et al., 2011), which require active virus replication, resulting in a greater susceptibility to subsequent infection by novel viruses such as H5N1 (Bodewes et al., 2010). With an increased appreciation of the immune response and its induction, it is time to consider new vaccine approaches for seasonal influenza that additionally or exclusively target functionally conserved regions of the influenza virus, and may therefore provide some level of disease reduction against serologically distinct emergent strains, even in a pandemic context.

IAVs, whose ancestral host is aquatic birds, have spread to many other species including domestic poultry, horses, swine, humans, and even fruit bats. Although there are 17 different distinct HA subtypes and 10 NA subtypes thus far identified for IAVs, only H1N1 and H3N2, and previously H2N2, subtypes have become endemic in humans causing continual human transmission and seasonal epidemics. The introduction of other novel subtypes, as exemplified by the H5N1, H7N9, and H10N8 strains, cause sporadic human infections and are not yet fully adapted for efficient human-to-human transmission. The segmented nature of the influenza virus genome facilitates reassortment to generate novel hybrid viruses between influenza viruses from different species, some of pandemic potential. Furthermore, the error-prone viral RNA-dependent RNA polymerase, which enables the generation of viral mutants, facilitates selection of influenza viruses resistant to anti-viral drugs and immune effectors. Thus, influenza is continually evolving and novel influenza viruses from animal reservoirs can cause unpredictable outbreaks, such as the most recent outbreaks from swine in the US (variant H3N2), poultry markets of China (H7N9) and Hong Kong (H5N1), leaving us unprepared and unprotected. Furthermore, H7N9 and H5N1 infections are highly lethal, with around 30 and 60% hospitalization-associated mortality, respectively. Therefore, there is a dire need for a vaccine that is effective against a “moving target,” influenza viruses.

## Cross-reactive antibodies lead to renewed interest in B cell vaccines

The novel 2009 H1N1 pandemic virus (pH1N1-09) spread worldwide within 4 months due to minimal specific Ab immunity across the population. However, due to the novelty of the HA protein in some cases infection or vaccination resulted in the induction of novel broadly “neutralizing” antibody responses (reviewed in Corti and Lanzavecchia, 2013), leading to a renewed interest in developing the targets of theses Abs (summarized in Table 1) for universal vaccines.

**Table 1:** **Potential targets for a universal influenza vaccine and their limitations**.

**Protein**	**Location**	**Site targetted**	**Function of target**	**Immune effector**	**Possible role in influenza protection**	**Escape possible**
HA	Virion surface	HAI_ (head)	Virus binding	nAb	Block HA binding	Yes
	Virion and cell surface	HA2 (stem)	Viral fusion	Non-nAb	Block HA maturation, Fc-mediated lysis	No
NA	Virion surface	Sialidase	Virus release	Non-nAb	Block NA cleavage and virus release	Yes
M2	Infected cell surface	M2 Ectodomain	Ion channel	Non-nAb	Block virus entry	No
NP	Infected cell surface	Unknown	RNP structure	Non-nAb	ADCC and complement mediated lysis	Unknown
	Infected cell	Conserved pMHC	RNP structure	T cells (CD4+ and CD8+)	T cell cytotoxicity reduces viral load	Yes (limited)
All	Infected cell	Conserved pMHC	Various	T cells (CD4+ and CD8+)	T cell cytotoxicity reduces viral load	Yes (limited)

**Figure 1:**
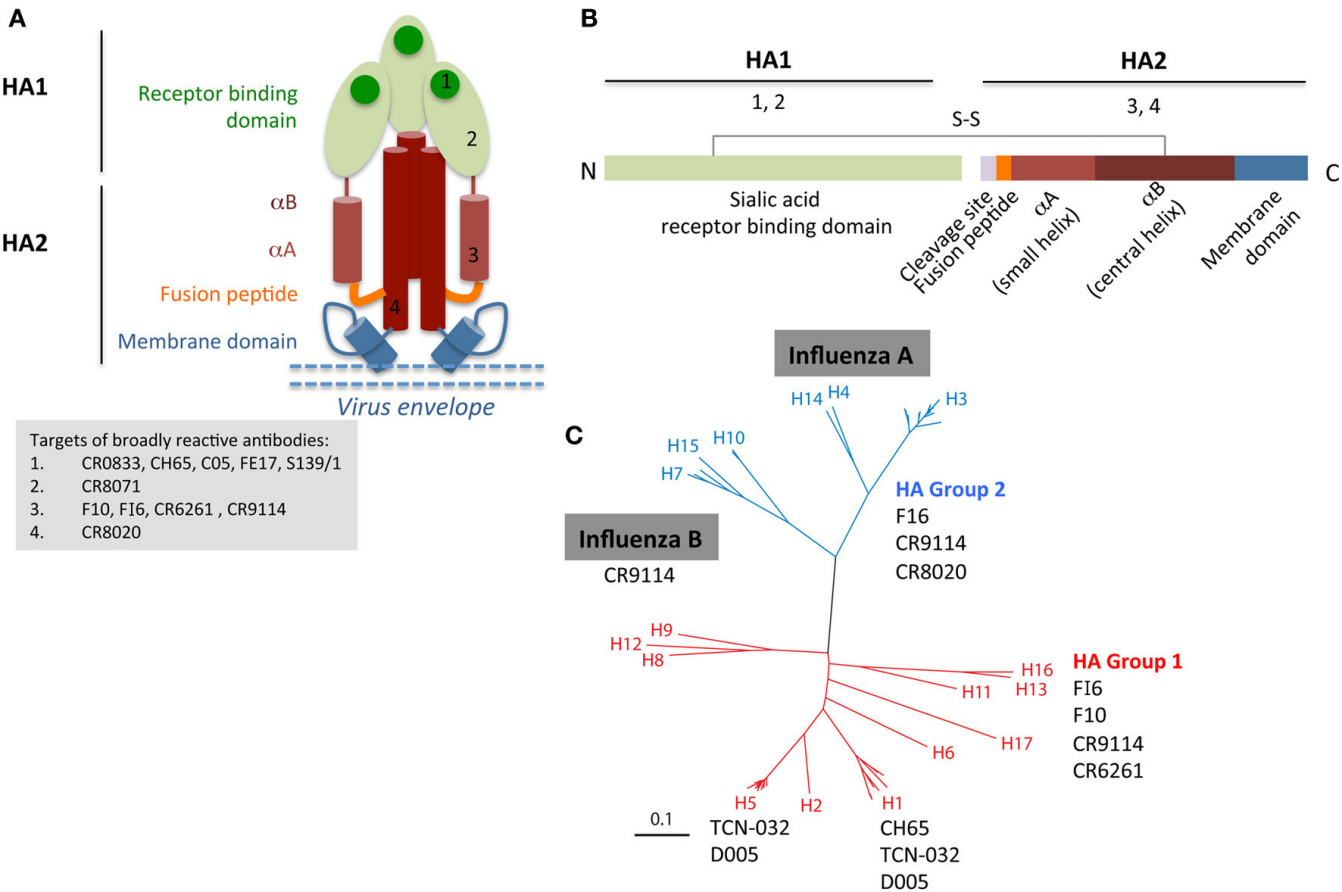
**Broadly reactive HA-specific Abs can bind different regions of the HA (A,B), which based on the sequence or structural conservation of the region targeted by Abs can lead to reactivity against otherwise highly divergent influenza viruses (C), such as influenza A group 1 and group 2 and even influenza B viruses.** The cartoon depiction of the HA protein **(A,B)** is not to scale. Group 1 and 2 phylogeny of influenza A HA **(C)** was adapted from Corti and Lanzavecchia (2013).

The HA glycoprotein on the influenza virion exists as a trimer and dominates the surface of the virus. The amino acid sequence shows 40–70% conservation between different HA subtypes (e.g. H1 vs. H7) and greater than 80% between strains within a single subtype (e.g. H1 strains). Subtype variation underlies the classification of influenza viruses into two phylogenetic groups: group 1 (H5, H2, H1) and 2 (H3, H7, H10). Each HA monomer consists of two disulphide-linked polypeptide chains HA1 and HA2 (Figures 1A,B). The majority of the HA1 chain goes to make up the globular head of the molecule, which contains the receptor-binding domain (RBD). The RBD is the primary target for nAb responses elicited by current vaccines, and therefore random mutations that lead to single amino acid changes in Ab binding sites in this domain are selected under pressure to avoid further such Abs, resulting in the process of antigenic drift. In some instances, amino acid changes that alter the glycosylation pattern in the head region can also influence Ab binding. Some bnAbs have been isolated that recognize epitopes on the HA head region, e.g. FE17 (Corti et al., 2010), S139/1 (Yoshida et al., 2009), CH65 (Whittle et al., 2011), C05 (Ekiert et al., 2012) (Figure 1). However, escape mutations in the HA1 can be generated either at Ab binding sites or flanking residues after several passages *in vitro*, thus limiting the use of head-specific bnAbs.

The stem of the HA, which supports the globular head, contains a hydrophobic fusion region which is situated at the N-terminus of the HA2 chain. This region plays a crucial role during viral entry into cells, allowing endosome escape of the viral genome, and is functionally and structurally conserved across HA subtypes and therefore not susceptible to drift. The stem contains a number of epitopes spanning the fusion region, which are conserved across different influenza subtypes (Okuno et al., 1993), enabling the isolation of influenza-specific broadly cross-reactive Abs capable of recognizing either group 1 HA (from Crucell CR6261) (Ekiert et al., 2009), group 2 HA (CR8020) (Ekiert et al., 2011), both group 1 and 2 HAs (FI6) (Corti et al., 2011), or even recognize different influenza A and B strains (CR9114) (Dreyfus et al., 2012) (reviewed in Corti and Lanzavecchia, 2013) (Figure 1). One hypothesis for the induction of post-pandemic group 1-specific stem Abs is that the pH1N1-09 virus displayed a sufficiently distinct HA1 head domain when compared to the pre-pandemic H1N1 viruses, that pre-existing memory B cells specific for the HA head could not be recruited stem-specific responses. Thus, H1N1-2009 virus exposure generated a primary HA1-specific H1 Ab response but in addition was able to boost the low frequency group 1 stem specific Abs (Corti et al., 2010; Margine et al., 2013).

Importantly, HA2 stem-specific Abs prevent the conformational changes required for viral entry and membrane fusion, thus mutational escape is not possible due to the critical function and conserved helical structure of the stem. However, very high concentrations of stem-specific Abs are often required to mediate virus neutralizing activity, as they are 100–1000 times less potent than HA head-specific Abs (Corti et al., 2010). Typically, stem-specific Abs do not inhibit sialic acid binding by the receptor-binding site, as do classical head-specific Abs. For this reason they are unable to be identified by standard haemaglutination inhibition (HAI) assays, and are measured by virus neutralization or modified ELISA type assays. It should also be noted that bnAbs often represent highly edited B cell receptor sequences from germline requiring affinity maturation and co-ordination with T follicular helper cells (reviewed in Corti and Lanzavecchia, 2013), and thus they are often very rare and of low frequency. Advanced B cell cloning techniques have enabled the identification and isolation of unique bnAbs (Corti et al., 2011) for prophylactic and therapeutic use.

Vaccination with the conserved HA2 stem was first explored as an Abs target nearly 30 years ago (Graves et al., 1983), and is now receiving renewed attention in the wake of the 2009 pandemic. Various methods have been employed by different research groups to elicit bnAbs following exposure to influenza, such as headless HA protein or virus exposing the stem (Steel et al., 2010; Wang et al., 2010), prime-boost with a chimeric HA (Krammer et al., 2013) or by sequential infection with different influenza subtypes (Krammer et al., 2012) and DNA-prime heterologous-boost vaccination (Wei et al., 2010). Furthermore, bnAbs (CR6261, CH65, and scF10) have even been incorporated directly into self-assembling nanoparticles, providing long-term passive immunity in animal models (Kanekiyo et al., 2013), a strategy that could potentially be beneficial for the elderly or immunocompromised. Importantly, using the murine model it has been recently elucidated that the protective effect of some broadly-neutralizing stem-specific Abs is dependent on FcγR interactions (DiLillo et al., 2014) so that their main activity is not by classical neutralization of virus particles.

Other broadly reactive Abs that work in this way are those directed to the highly conserved surface M2 ion channel ectodomain (M2e). M2 is only expressed in very small amounts on the virion surface but is present on the infected cell surface where it can form a target for cross-reactive lytic responses. M2e-specific Abs are induced only at very low frequency by influenza infection, and are not elicited at all by the standard TIV vaccination, although they can be induced by M2e-specific vaccines (Neirynck et al., 1999; Mozdzanowska et al., 2003). Unexpectedly, Abs specific for the viral nucleoprotein (NP), which surrounds the genome, have also shown passive protection in mouse models at very high doses (Carragher et al., 2008; Lamere et al., 2011) and can be found in human serum (Sukeno et al., 1979). *In vitro* studies demonstrated that influenza-infected cells express low levels of NP on their surface (Virelizier et al., 1977; Yewdell et al., 1981), which may enable NP recognition by immune effectors, or alternatively, it is possible that NP-specific Abs are internalized and interrupt virus replication. Utilization of non-neutralizing NP and M2e Abs might be beneficial when combined with additional protective immune mechanisms.

Abs that are not virus neutralizing may also function in Ab-dependent cellular cytotoxicity (ADCC). pH1N1-09, H5N1-specifc and NP-specific ADCC Abs have been found in the absence of nAb responses in healthy individuals (Jegaskanda et al., 2013b). Influenza infection, but not standard TIV vaccination of macaques, elicited H1N1-specific ADCC Ab responses (Jegaskanda et al., 2013a), thus future vaccines would need to be optimized to elicit ADCC responses.

However, a forewarning comes from recent evidence in mouse models, which showed that influenza virus was able to specifically infect influenza-specific B cell receptor (BCR)-expressing B cells leading to BCR editing, thus allowing establishment of viral infection despite pre-existing Ab responses (Dougan et al., 2013). There is also evidence from a swine vaccination model that stem-specific HA2 Abs can enhance viral fusion and increase immunopathology upon H1N1pdm infection (Khurana et al., 2013). Therefore, while broadly cross reactive Abs are an increasingly promising area for combating influenza infections of distinct strains, their use should not be without investigation and should be used in conjunction with additional immune mechanisms.

## Heterosubtypic T cell responses for influenza

CD8^+^ T cells recognize virus-derived peptides in the context of class I major histocompatibility antigens (MHC-I). pMHC-I is displayed on the surface of APCs enabling CD8^+^ T cell priming and on virus-infected cells for CD8^+^ T cell effector function, thus infected cells can be killed before virus progeny is released. The cytotoxic function is mediated mainly via the delivery of perforin and granzymes into the infected cell (Topham et al., 1997) as well as by cytokine release (Marshall et al., 2005). Thus, CD8^+^ T cell recognition of influenza viruses is only possible for an established infection, in contrast to sterilizing nAb responses. However, CD8^+^ T cells are critical in the elimination of influenza viruses, expediting viral clearance, and reducing pathology. Seminal work from influenza challenge of healthy human volunteers showed that increased T cell cytotoxicity was associated with reduced virus shedding (McMichael et al., 1983b), even in volunteers lacking nAbs against the infecting virus. Moreover, high levels of influenza-specific pre-existing memory T cells have been associated with milder symptoms during pH1N1 infection (Sridhar et al., 2013). There is no doubt that the current Ab-based approach should be maintained, but the incorporation of an even far-from-perfect T-cell-inducing vaccine (or vaccine component) could still save millions of lives during a pandemic, as T cells have the potential for much broader protection than bnAbs across diverse subtypes of influenza A.

The phenomenon of heterosubtypic immunity refers to memory T cells generated by one subtype that can cross-react against different IAV subtypes, despite wide differences in surface glycoproteins (Braciale, 1977; Kees and Krammer, 1984; Yewdell et al., 1985; Askonas et al., 1988; Wahl et al., 2009). T cell heterosubtypic immunity is mainly due to the majority of T cells recognizing immunogenic peptides derived primarily from highly conserved internal influenza proteins (Elhefnawi et al., 2011). For CD8^+^ T cells, 193 immunogenic peptides presented by 51 different Human Leukocyte Antigen Class I (HLA-I) molecules have been described to date for influenza viruses (www.iedb.org). The majority of CD8^+^ T cell antigenic peptides are derived from well-conserved internal proteins NP, M1, and PB1 (Assarsson et al., 2008; Lee et al., 2008; Wu et al., 2011; Grant et al., 2013). T cell cross-reactivity between different IAVs has been demonstrated between H1N1, H3N2, H2N2, H5N1, H3N2v, and H7N9 subtypes (Table 2) (Epstein, 2006; Kreijtz et al., 2008; Greenbaum et al., 2009; Hillaire et al., 2013; Quinones-Parra et al., 2014; Van De Sandt et al., 2014). Thus, influenza-specific T cells can provide universal protection against influenza disease and are of significant interest for the design of next generation vaccines.

**Table 2:** **Key human studies on cell-mediated immunity against IAV**.

**References**	**Virus(es)**	**Description**
McMichael et al., 1983b	H1N1	Lymphocyte cytotoxic activity was associated with lower virus shedding in individuals challenged with H1N1
Gotch et al., 1988	H3N2→H1N1	CD8^+^ T cell lines generated with H3N2 virus lyse target cells infected with Vaccinia viruses encoding NP, M1, or PB2 proteins derived from H1N1
Epstein, 2006	H1N1→H2N2	Adults that contracted H1N1 influenza prior to the emergence of pH2N2-57 were pronouncedly less susceptible to the pandemic virus
Kreijtz et al., 2008	H3N2→H5N1	CD8^+^T cell lines established with H3N2 cross-react with immunogenic peptides derived from H5N1
Lee et al., 2008	Seasonal (s) IAV→H5N1	CD4^+^ and CD8^+^ T cells from H5 seronegative donors respond to peptides spanning the H5N1 proteome
Assarsson et al., 2008	H1N1, H3N2, H2N2, H5N1, H7N7, H6N1, H7N7, and H9N2	CD4^+^ and CD8^+^ T cells from healthy donors respond to substantially conserved immunogenic peptides
Tu et al., 2010	sH1N1/sH3N2→pH1N1-09	Purified, influenza-specific memory CD8^+^ T cells expanded with sH1N1 and sH3N2 recognize target cells infected with pH1N1-09
Gras et al., 2010	pH1N1-09→pH1N1-1918	B7-NP_418_-specific CD8^+^ T cells elicited by pH1N1-09 infection cross react with the pH1N1-1918-NP_418_ variant
Wilkinson et al., 2012	H3N2	Pre-existing CD4^+^ T cell responses correlated with lower virus shedding and disease severity upon challenge with H3N2 in seronegative volunteers
Fox et al., 2012	pH1N1-09	CD8^+^ T cell activation is delayed in patients severely infected with pH1N1-09 and are lymphopenic for CD4^+^, CD8^+^ T cells, and NK cells
Zhao et al., 2012	pH1N1-09	Influenza-specific CD4^+^ T cells responses are associated with progression to severe pH1N1-09 infection
Sridhar et al., 2013	pH1N1-09	Pre-existing memory CD8^+^ T cell responses from seronegative patients naturally exposed to pH1N1-09 correlate with reduced illness severity
Hillaire et al., 2013	sH1N1/sH3N2→pH1N1-09/H3N2v	CD8^+^ T cells lines generated with sH1N1, sH3N2 viruses or peptides derived from these strains respond to target cells infected with pH1N1 virus
Van De Sandt et al., 2014	pH1N1/sH1N1/sH3N2→H7N9	CD8^+^ T cells stimulated with sH1N1, sH3N2, or pH1N1 recognize and lyse target cells infected with H7N9
Quinones-Parra et al., 2014	Any human IAV including H7N9	CD8^+^ T cells from healthy donors expressing the HLA-A*0201, -A*0301, -B*5701, -B*1801 allele, and/or B*0801 allele(s) respond to universally conserved immunogenic peptides

The viral clearing role of heterosubtypic killer CD8^+^ T cells is well established in animal models and human studies (Table 2). In a primary infection of CD8^+^ T cell-deficient mice, influenza virus clearance is delayed and mortality is increased (Bender et al., 1992), while in the absence of B cells or Abs, CD8^+^ T cells can provide protection against otherwise lethal influenza (Graham and Braciale, 1997; Epstein et al., 1998). Furthermore, transfer of influenza-specific CD8^+^ T cells provides heterosubtypic protection (Yap et al., 1978; Taylor and Askonas, 1986). Secondary recall responses from pre-existing memory CD8^+^ T cells, established by either influenza virus infection or vaccination (Flynn et al., 1999) resulted in superior viral clearance and reduced pathology (Flynn et al., 1998). Moreover, tertiary challenge of mice with highly lethal H7N7 resulted in recall of heterosubtypic memory CD8^+^ T cell responses (established from priming with H1N1 and then H3N2) that provided exceptionally enhanced virus control (within 3 days post-infection) (Christensen et al., 2000).

In comparison to CD8^+^ T cells, the role of CD4^+^ T cells in influenza is less well understood, partly due to their heterogeneity and the lack of epitope-specific systems (Sant and McMichael, 2012). The traditionally accepted role of influenza-specific CD4^+^ T cells is in providing help to B cells for the production of high-quality Abs (Topham and Doherty, 1998), as their activation is dependent on recognition of peptide in the context of MHC-II on professional APCs but also have a major role in providing help for the establishment of CD8^+^ T cell memory, critical for a robust recall response (Sun et al., 2004). Transfer of influenza-specific effector CD4^+^ T cells into T cell-deficient mice accelerates production of neutralizing Abs, thus cross-reactive memory CD4^+^ T cells can potentially enhance B cell responses during infection with a novel influenza virus (Scherle and Gerhard, 1986). Furthermore, depletion of CD4^+^ T cells prior to influenza challenge results in a dramatic drop of Ab titres (Eichelberger et al., 1991), accompanied by only a small delay in virus elimination (Allan et al., 1990), driven by the remaining CD8^+^ T cell response (Topham et al., 1996; Belz et al., 2002). More recently, a comprehensive transgenic mouse study by McKinstry et al. (2012) illustrated the direct protective role of influenza-specific CD4^+^ T cells using a series of transfer experiments into immune knockout mice (lacking FcRγ, functional IFNγ, or B cells). The authors showed that CD4^+^ T cells provide protection by interacting with B cells and CD8^+^ T cells in an IFN-γ-dependent manner. The mechanism by which CD4^+^ T cells are able to recognize virus-infected cells given their MHC-II restriction is yet to be deciphered. Nevertheless, in a vaccination setting of individuals receiving a split vaccine, a subset (ICOS^+^CXCR3^+^CXCR5^+^) of circulating influenza-specific CD4^+^ T follicular helper (T_FH_) cells correlated with more effective B-cell responses and greater Ab titres, suggesting that eliciting this type of cells could be important in inducing more effective Ab-based vaccines (Bentebibel et al., 2013).

The debate of which T cell subtype is more protective for influenza has been reinvigorated from recent human studies that show contrasting results (Wilkinson et al., 2012; Zhao et al., 2012; Sridhar et al., 2013). However, as evidenced from mouse studies outlined above, both subsets are necessary for a complete and coordinated response against influenza infection. Interestingly, in a challenge study (Wilkinson et al., 2012) with volunteers deliberately infected with H3N2, the numbers of pre-existing influenza-specific CD4^+^, but not CD8^+^, T cells were found to correlate with lower virus shedding and less severe, shorter illness. These investigators favored CD4^+^ T cell cytotoxicity as the possible underlying mechanism. However, in a later study investigating T cell immunity in H1N1pdm virus-infected patients, CD4^+^ T cell responses were associated with more severe infection (Zhao et al., 2012). A more recent study (Sridhar et al., 2013) followed natural infection of a large cohort over the 2009 pandemic and determined pre-immune correlates with the outcome of influenza disease over the pandemic. The study showed that those individuals with established influenza-specific CD8^+^ T cell memory experienced milder illness following infection with the newly emerged virus. Although this study did not find a correlation with CD4^+^ T cell responses and disease outcome, it cannot rule out their importance. Further studies are needed to better understand immune mechanisms underlying T cell-mediated protection against influenza viruses.

Some experimental vaccination protocols have effectively induced protective heterosubtypic T cell immunity, including non-replicative, cold adapted influenza vaccine (Powell et al., 2007), and virus-like particles (Hamada et al., 2013). In addition, DNA vaccines (Ulmer et al., 1993, 1998; Fu et al., 1999), prime-boost protocols (Epstein et al., 2005) and the use of adjuvants can provide and enhance T cell-mediated heterosubtypic protection (Chua et al., 2011, 2014). A live non-replicating vaccinia vaccine encoding the NP and M1 proteins, MVA-NP/M1, is currently being evaluated in human efficacy trials (Berthoud et al., 2011). The vaccine proved effective for human influenza challenge in a limited number of individuals, showing higher levels of influenza-specific CD8^+^ T cells in vaccinees, especially those displaying the HLA-A^*^0201 allele, compared to placebo controls correlating with reduced infection and viral shedding (Antrobus et al., 2012; Lillie et al., 2012). Furthermore, the vaccine was able to boost T cell immunity in those aged 65 and over, a promising result for those who need an effective vaccine the most. However, it may be difficult to convince regulatory authorities to license a T cell-based vaccine that still allows individuals to become infected and shed virus. If such a vaccine were to replace the current TIV, a large-scale study would need to be performed in many individuals of distinct HLAs across different ethnicities to prove effectiveness.

Thus, manipulating existing style vaccines to induce or boost T cell immunity could potentially lead toward development of broadly protective influenza vaccines. However, there are still considerable challenges in the development of broadly cross-reactive T cell-inducing vaccines, which include persistence of T cell memory after influenza immunization (Valkenburg et al., 2012), population protective coverage across different HLAs, vaccine-mediated immune escape and immunopathology. Firstly, it is still far from clear for how long functional influenza-specific memory CD8^+^ T cells persist in humans. Studies from yellow fever and smallpox vaccination suggest that memory T cells can be detected from 10 years (Akondy et al., 2009) to 50 years (Miller et al., 2008) following vaccination, respectively. Yet many adults fail to control influenza infection. A vaccine study of young children found that a threshold level (of >100 SFU/10^6^ PBMCs) was required for effective T cell-mediated clinical protection (Forrest et al., 2008). Therefore, the varying levels of T cell immunity that are likely to exist in the wider population, due to different histories of exposure to natural infection, may contribute to the spectrum of disease severity. Early studies on cytotoxic T cells in humans suggested that influenza CTL memory declines rapidly with a half life of 2–3 years (McMichael et al., 1983a). The main purpose of a T cell-inducing vaccine may therefore be to maintain memory CD8^+^ T cells at levels capable of achieving clinical protection, which may require booster doses every few years. The presence of co-morbidities, age-related differences of innate responses in the young and immunological decline in the elderly (reviewed by Oshansky and Thomas, 2012) could also impair T cell recall responses. Furthermore, an influenza T-cell based vaccine would have to address the issue HLA coverage in a diverse population and be appropriate for ethnic minorities with rare MHC alleles. This could be achieved either by utilization of peptide epitopes representing HLA-super families (Assarsson et al., 2008), or the inclusion of full-length influenza-derived proteins in a form that enables endogenous antigen processing.

## Influenza can escape T cell immunity

If such a T cell-inducing vaccine could be produced, the issue of vaccine-mediated T cell escape, resonant of Ab-mediated antigenic drift, could theoretically become significant. RNA viruses, such as influenza, are characterized by poor fidelity of replication of their genomes, leading to the emergence of viral variants capable of rapidly adapting in response to immune selective pressure, as seen with seasonal antigenic drift. Subversion of T cell control is well documented for chronic viral infections, like HIV and HCV (Pircher et al., 1990; Moore et al., 2002; Fernandez et al., 2005) and represents one of the major obstacles for viral control and vaccine design.

Within an individual, T cells can select influenza escape variants as the virus replicates. We have recently described the emergence of influenza variants within CD8^+^ T cell target regions in a persistently infected, immunocompromised child, (Valkenburg et al., 2013). Additionally, CD8^+^ T cell immune escape viruses could be readily isolated from immunodeficient (B cell knockout) and immune intact wild-type mice. Surprisingly, we observed that these CTL escape variants arise early during infection by day 5, and increase in frequency and variety over the time-course of infection. The selection of CD8^+^ T cell escape mutants was clearly driven by selective pressure as these variants revert in the absence of immune pressure in MHC-mismatched mice (Valkenburg et al., 2013). Further experiments suggested that influenza-specific escape from T cell responses is heavily dependent on the particular epitope and potentially the underlying characteristics of the T cell receptor repertoire. Interestingly, influenza viruses favored escape at the residues that anchor epitope peptides to MHC (Valkenburg et al., 2013).

The emergence of specific CD8^+^ T cell mutations in influenza can also be detected at a population level. Factors such as immunodominance, HLA frequency and viral fitness can impact the likelihood of emergence and selection of escape viruses (Berkhoff et al., 2005). Indeed, there are documented examples of naturally occurring mutations within critical T cell antigenic peptides leading to immune escape in individuals bearing certain HLA alleles. The Rimmelzwann group has pioneered and enhanced our knowledge of CD8^+^ T cell-mediated immune escape in human influenza. Boon et al. (2004) identified substitutions within the HLA-B^*^0702- and B^*^3501-restricted NP_418–426_ epitope that could either result in cross-reactivity, or in some cases, in immune escape. Further immunological and structural characterization of the NP_418–426_ variants from 1918 to 2009 revealed that mutations in solvent-exposed, potentially TCR contact residues, result in immune escape (Gras et al., 2010). At the same time, we confirmed the presence of cross-reactive CTL populations that reacted against a wider spectrum of NP_418–426_ variants (Gras et al., 2010). The identification of epitopes recognized by such populations, and the key residues for TCR recognition within them, may be critical information for producing a vaccine capable of eliciting cross-reactive T cells to provide coverage against the wide spectrum of influenza antigenic variation, an idea that we have pioneered using the B6 mouse model of influenza infections (Valkenburg et al., 2013). Conversely, CD4^+^ T cell-mediated viral escape in influenza has received considerably less attention and the selection of CD4^+^ T cell escape peptide variants has not currently been demonstrated, either within an individual or across a population.

In earlier work examining drift in the viral NP at a population level, Voeten et al. (2000) identified mutations in HLA-B^*^2705-restricted NP_383–391_ epitope and HLA-B^*^08:01-NP_380–388_ resulting in immune escape. The NP-R384K mutation at an MHC-I anchor residue, which resulted in a loss of CD8^+^ T cell recognition, was initially detected in 1990 and later in 1993 as R384G. The escape mutant quickly replaced the wild-type sequence in H3N2 viruses (Gog et al., 2003), resulting in a loss of immunogenicity in the population expressing the HLA-B27 allele. Further characterization of this mutant indicated that the escape was driven by CTL selective pressure as the mutation imposed a fitness cost that had to be compensated for by additional mutations in flanking regions (Rimmelzwaan et al., 2005). More recently, a CTL escape variant within the HLA-A^*^0101-restricted NP_44–52_ in the novel H7N9 virus has been reported (Quinones-Parra et al., 2014; Van De Sandt et al., 2014), with structural data indicating that substitution in an MHC-I anchor residue of the peptide epitope compromised peptide-MHC-I complex stability and thus accessibility to CTLs (Quinones-Parra et al., 2014).

Although immunodominant T cells may generate escape variants, they remain a hugely valuable tool in the arsenal for combating influenza infection. It may be possible to pre-empt escape by priming the T cell repertoire against a variety of dominant mutants at TCR contact sites (Valkenburg et al., 2013) or possibly stabilizing the MHC for low affinity anchor mutants. The NP protein, though capable of harboring T cell escape mutations, is one of the most immunogenic influenza proteins for T cells (Grant et al., 2013). Fortunately, the NP of LAIV can be substituted to represent the current NP or the NP of escape variants without affecting the viral growth or vaccine immunogenicity (Isakova-Sivak et al., 2011).

## Innate T cells for influenza virus infection

Until now this review has discussed adaptive immunity to influenza, however another component, innate T cells, have potential use for subverting infection due to innate receptors recognizing conserved universal motifs. The non-conventional or innate T cell compartment comprises of γδ T cells, CD1d-restricted invariant natural killer T cells (iNKT), and MR1-restricted Mucosal-associated invariant T (MAIT) cells. This compartment constitutively expresses high levels of the C-type lectin, CD161. Innate T cells can be activated by a diverse range of ligands, either endogenous (β-GlcCer and iGB3 for NKT; MICA/B for γδ T cells) and exogenous (phosphoantigens and bisphosphonates for γδ T cells, α-GalCer-for iNKT, bacterial lipids for NKT and metabolites for MAIT cells) (Kjer-Nielsen et al., 2012; Rossjohn et al., 2012; Born et al., 2013). Upon TCR recognition of their cognate antigen or by cytokine driven signals, innate T cells can rapidly produce an array of inflammatory and effector molecules (IFNγ, TNF, IL-17, IL-4, IL-22, perforin, granzyme B, MIP-1β). In humans, innate T cells can comprise up to 30% of the peripheral blood CD3^+^ T cell compartment, and are also enriched at mucosal sites including lung, intestine and liver. Given their location, potent inflammatory and cytolytic function, innate T cells are potentially important players during IAV infection. Although their role is well studied in autoimmunity, cancer, and chronic viral infections such as HIV-1, (Berzins et al., 2011; Cosgrove et al., 2013; Leeansyah et al., 2013; Vantourout and Hayday, 2013), there is a paucity of data on how innate T cells contribute to combating influenza infection.

Several studies in humans have demonstrated that a major subset of human γδ T cells (Vγ9Vδ 2) can directly kill human and avian origin influenza-infected macrophages and lung alveolar epithelial cells *in vitro* (Qin et al., 2009; Tu et al., 2011; Li et al., 2013). Further studies in humanized mice have shown that vaccination with aminobisphosphonate pamidronate (PAM) can activate the Vγ9Vδ 2 subset, and that Vγ9Vδ 2 T cells can inhibit viral replication and dampen inflammatory responses in the lung (Tu et al., 2011). Thus, pre-arming γδ T cells may also be beneficial in human IAV infection as an alternative antiviral strategy (Tu et al., 2011).

Similarly, endogenous iNKT cells have been shown to have an immunoregulatory role in IAV. From adoptive transfer studies in C57BL/6 mice, iNKT cells have been demonstrated to alleviate bronchopneumonia and its associated pathology in Jα18^−/−^ (iNKT deficient) mice infected with highly virulent H3N2 (Paget et al., 2011; Kok et al., 2012). Both endogenous and exogenous (α-GalCer) activation of iNKT aids in the development of influenza-specific CD8^+^ T cells by promoting their survival (Guillonneau et al., 2009) and the maturation of APCs that present epitopes to influenza-specific CD8^+^ T cells (Paget et al., 2011). Additionally, iNKT-derived cytokines, such as IL-33 and IL-22, produced during IAV infection have been associated with regulation of eosinophil maturation in an IL-5-dependent manner and protection of the airway epithelium, respectively (Paget et al., 2012; Gorski et al., 2013). These studies demonstrate that iNKT can aid in the maturation of both the influenza-specific adaptive and the innate response, and therefore may be important subsets to induce in a universal IAV vaccine.

Although the majority of these studies highlight the immunoregulatory role of murine innate T cells in models of IAV, more studies are needed in humans to investigate whether these and other non-conventional innate T cells may contribute to influenza-specific adaptive immune effectors and thus be of benefit to induce in a universal IAV vaccine.

## Conclusions

Although the current Ab-mediated vaccines are the most cost effective way to combat the yearly influenza epidemics, they are strain-specific and thus need to be updated annually while providing little or no protection from novel outbreak strains. Furthermore, during the 2009 pandemic, it took several months to produce and test the newly made H1N1pdm-specific influenza vaccine, which meant it was only available after the peak of influenza activity. Thus, there is an urgent need to develop novel approaches for a universal influenza vaccine that has broad reactivity across a diversity of influenza strains and subtypes. Ideally, this vaccine would elicit both broadly cross-reactive Abs directed at highly conserved yet sub-dominant targets such as the HA stalk, which are currently proving highly effective in mouse studies. Furthermore, the ideal vaccine would also elicit a robust T cell response with long-term memory potential, recognizing epitopes derived from conserved and immunogenic internal proteins. Recent data on both universal Abs and T cell responses against influenza are promising, but more research still needs to be done to provide insights into the longevity and effectiveness of this type of immunity. The possibility of generating escape mutants by widespread use of vaccines designed to elicit such cross-reactive responses needs to be understood as well as the potential impact on virus evolution.

### Conflict of interest statement

The authors declare that the research was conducted in the absence of any commercial or financial relationships that could be construed as a potential conflict of interest.
